# A Meta-Analysis of Early, Mid-term and Long-Term Mortality of On-Pump vs. Off-Pump in Redo Coronary Artery Bypass Surgery

**DOI:** 10.3389/fcvm.2022.869987

**Published:** 2022-04-25

**Authors:** Shicheng Zhang, Siyuan Huang, Xieraili Tiemuerniyazi, Yangwu Song, Wei Feng

**Affiliations:** Department of Cardiac Surgery, Fuwai Hospital, Chinese Academy of Medical Sciences and Peking Union Medical College, Beijing, China

**Keywords:** redo coronary artery bypass grafting, on-pump, off-pump, mortality, comparison

## Abstract

We aimed to compare the early, mid-term, and long-term mortality between on-pump vs. off-pump redo coronary artery bypass grafting (CABG). We conducted a systematic search for studies comparing clinical outcomes of patients who underwent on-pump vs. off-pump redo CABG. We pooled the relevant studies quantitatively to compare the early (perioperative period, whether in hospital or within 30 days after discharge), mid-term (≥1 year and <5 years), and long-term (≥5 year) mortality of on-pump vs. off-pump redo CABG. A random-effect model was applied when there was high heterogeneity (I^2^ > 50%) between studies. Otherwise, a fixed-effect model was utilized. After systematic literature searching, 22 studies incorporating 5,197 individuals (3,215 in the on-pump group and 1,982 in the off-pump group) were identified. A pooled analysis demonstrated that compared with off-pump redo CABG, on-pump redo CABG was associated with higher early mortality (OR 2.11, 95%CI: 1.54–2.89, *P* < 0.00001). However, no significant difference was noted in mid-term mortality (OR 1.12, 95%CI: 0.57–2.22, *P* = 0.74) and long-term mortality (OR 1.12, 95%CI: 0.41–3.02, *P* = 0.83) between the two groups. In addition, the complete revascularization rate was higher in the on-pump group than the off-pump group (OR 2.61, 95%CI: 1.22–5.60, *P* = 0.01). In conclusion, the off-pump technique is a safe and efficient alternative to the on-pump technique, with early survival advantage and similar long-term mortality to the on-pump technique in the setting of redo CABG, especially in high-risk patients.

**Systematic Review Registration:**

https://www.crd.york.ac.uk/PROSPERO/, identifier: CRD42021244721.

## Introduction

For more than 50 years, coronary artery bypass grafting (CABG) has been the surgical strategy for severe coronary artery disease (1). Nowadays, CABG is the most common cardiac surgery (2) and several techniques have been developed to improve the surgical outcomes (3). Extracorporeal circulation was first conceived of by Gibbon and after over 7 decade improvement has now been extensively utilized in cardiac surgery including CABG (on-pump CABG) (4). CABG without cardiopulmonary bypass (off-pump CABG) was first attempted by Goets in 1960 and is applied as commonly as on-pump CABG in some experienced cardiac centers nowadays (5). Numerous studies has investigated the outcomes of the on-pump vs. off-pump in the primary CABG (6). With an increasing number of patients undergoing CABG and a longer time after CABG, more and more patients suffered from recurrence of angina pectoris short or long after primary CABG. Most of these patients could be managed through percutaneous coronary intervention to relieve the angina pectoris (7). However, a small portion of them may require repeat revascularization through surgery, that is, redo CABG. Several studies compared the clinical outcomes of on-pump and off-pump in redo CABG. Among the numerous clinical outcomes, all-cause mortality is about the biggest concern of medical care providers as well as the patients. Therefore, we performed a meta-analysis of early, mid-term and long-term mortality of on-pump vs. off-pump in redo CABG.

## Methods

All comparative studies of on-pump vs. off-pump redo CABG published until 11 January 2021 were identified *via* systematic searches using the following databases: Pubmed, Embase and Web of Science. The search was supplemented by a manual search of references of initially identified articles. The search strategy included the key term of “coronary,” “redo,” “repeat,” “revascularization,” “pump,” “mortality.” Two reviewers assessed all identified articles independently, while a third reviewer was consulted to resolve the disputes. The publication language was restricted to English. This meta-analysis was performed in accordance to the Preferred Reporting Items for Systematic Reviews and Meta-analyses (PRISMA) guidelines. The research has been registered at the international prospective register of systemic review (PROSPERO) and register ID is CRD42021244721.

Studies for inclusion should meet the following criteria: the design was a comparative study; patients in the study were those who underwent redo CABG; patients were assigned to on-pump or off-pump group; outcomes should include early (perioperative period, in hospital or 30-day), midterm (≥1 year and <5 year) or long-term (≥5 year) all-cause mortality. Exclusion criteria: studies without comparison or not in English; studies with insufficient data of mortality; case reports, conference abstract, editorial or review.

Two authors independently extracted data including: basic information of the studies, demographic characteristics, mortality or survival rate, complete revascularization rate and perioperative neurological event rate. Discrepancy was discussed and resolved by consulting a senior author.

All the included articles were reviewed in detail independently by two reviewers. The Newcastle Ottawa Quality Assessment Scale was used to assess article's quality. The assessment scale consists of three parts: selection, comparability, and exposure, with eight items. The combined score ranges from zero to nine stars. We rated articles with seven to nine stars as high quality, five to six stars as medium quality, and zero to four stars as poor quality. Articles with poor quality were excluded. In addition, funnel plots were applied to detect publication bias.

All analyses were conducted using Review Manager version 5.3. Study-specific estimates were pooled using inverse variance method. Odds ratio (OR) were calculated for dichotomous variables, and reported with 95% confidence intervals (CIs). A *P*-value of <0.05 was considered statistically significant. Determination of heterogeneity was undertaken using the *I*^2^ value with *I*^2^ <50, 50–75, >75% denoting a low, moderate, high degree of statistically significant heterogeneity, respectively. If there was high degree of heterogeneity among studies, a random-effect model was used for calculating pooled effect. Otherwise, the fixed-effect model was utilized. In addition, sensitivity analyses were conducted by excluding studies one by one.

## Results

**Figure 1:**
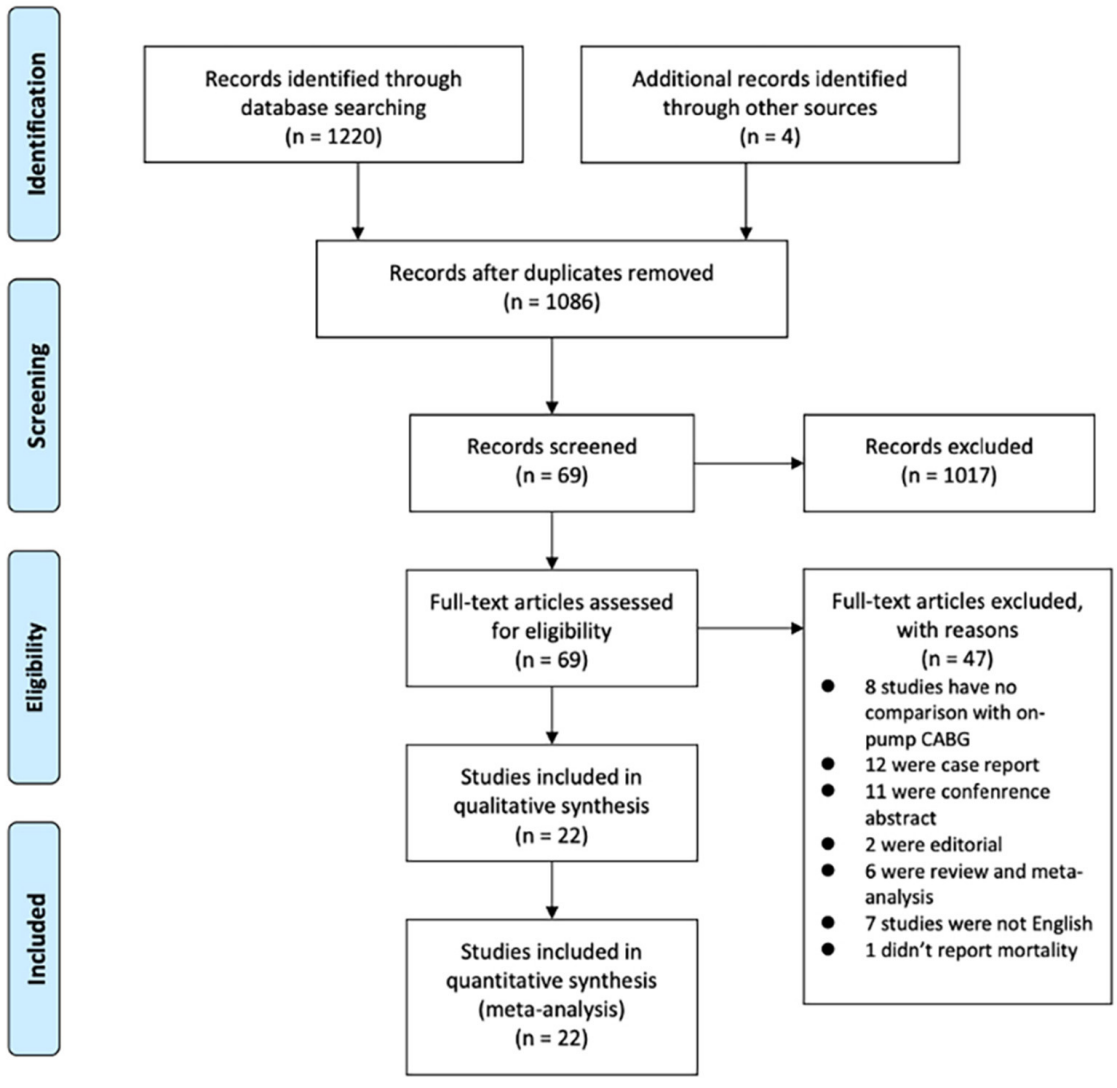
Preferred Reporting Items for Systematic Reviews and Meta-analyses (PRISMA) study selection flow diagram.

**Figure 2:**
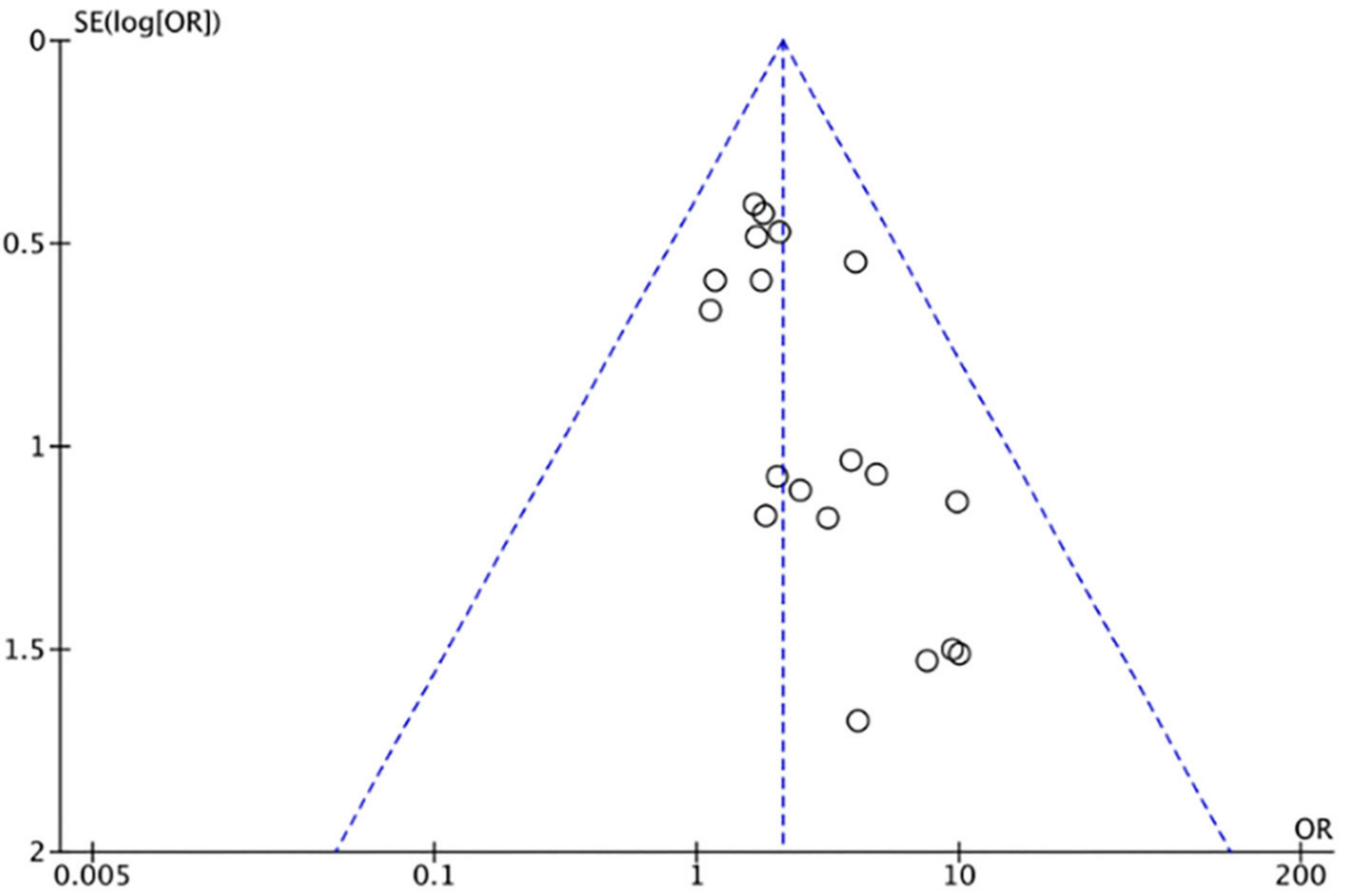
Funnel plot for early mortality.

**Figure 3:**
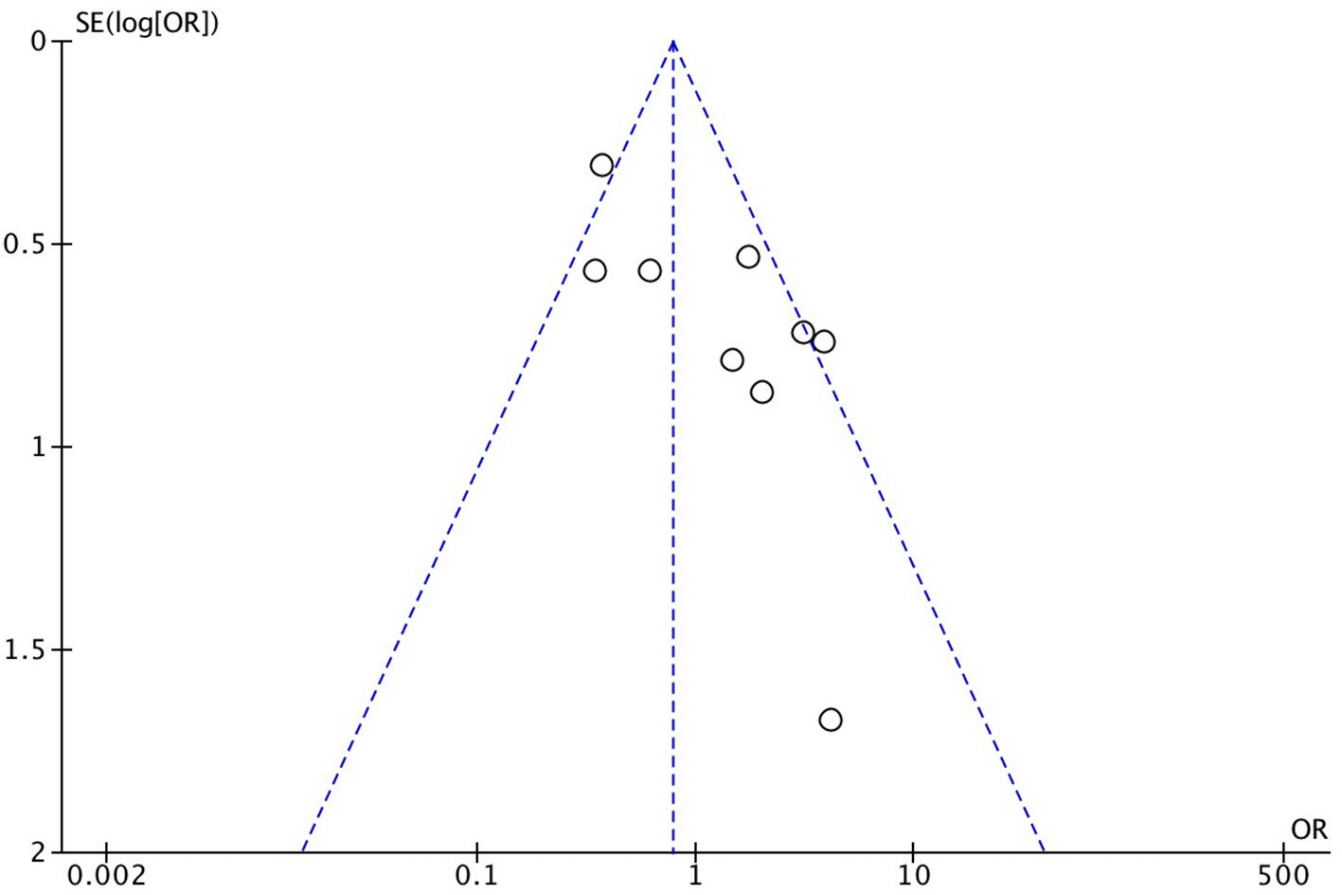
Funnel plot for mid-term mortality.

**Figure 4:**
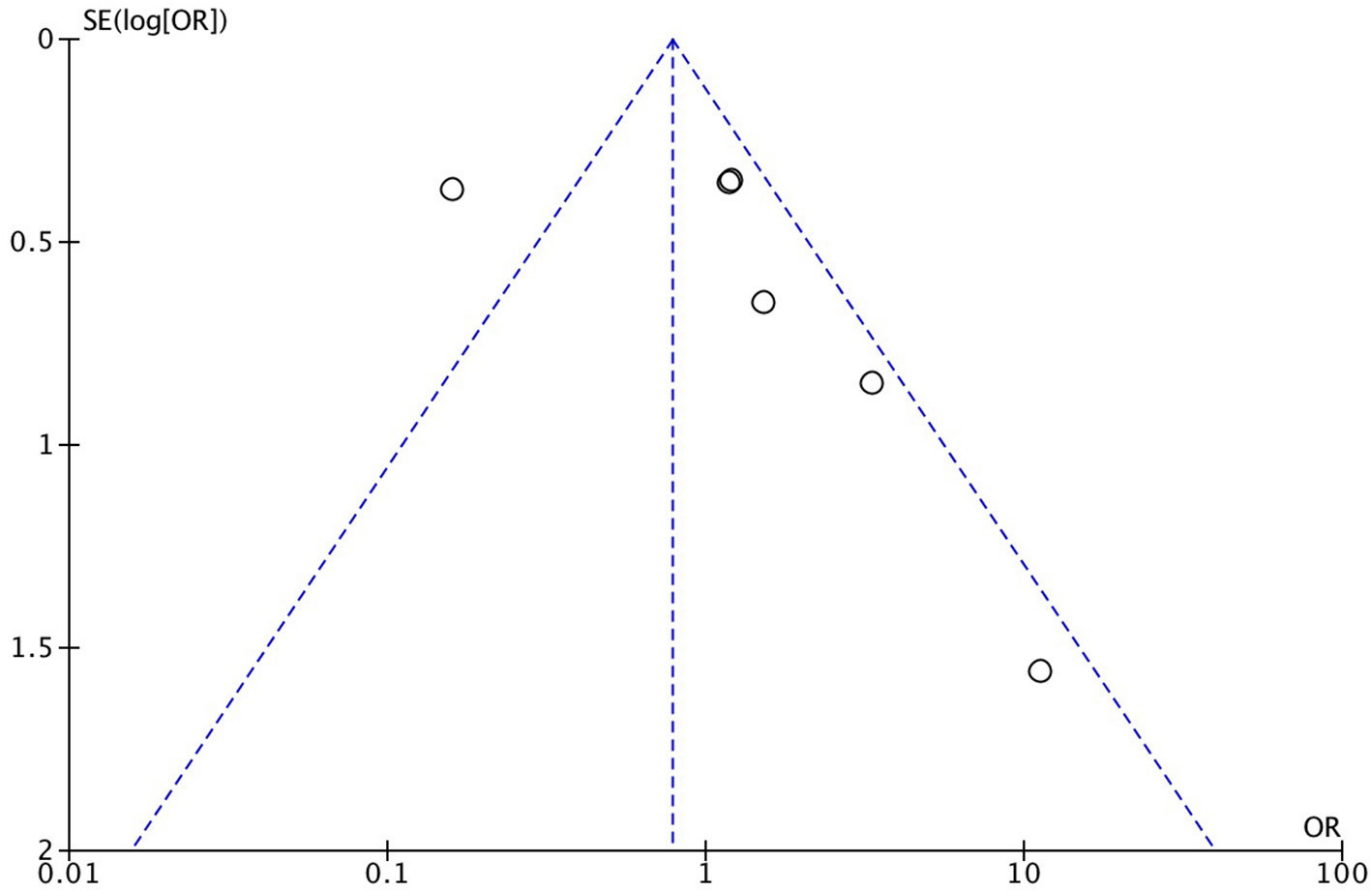
Funnel plot for long-term mortality.

**Figure 5:**
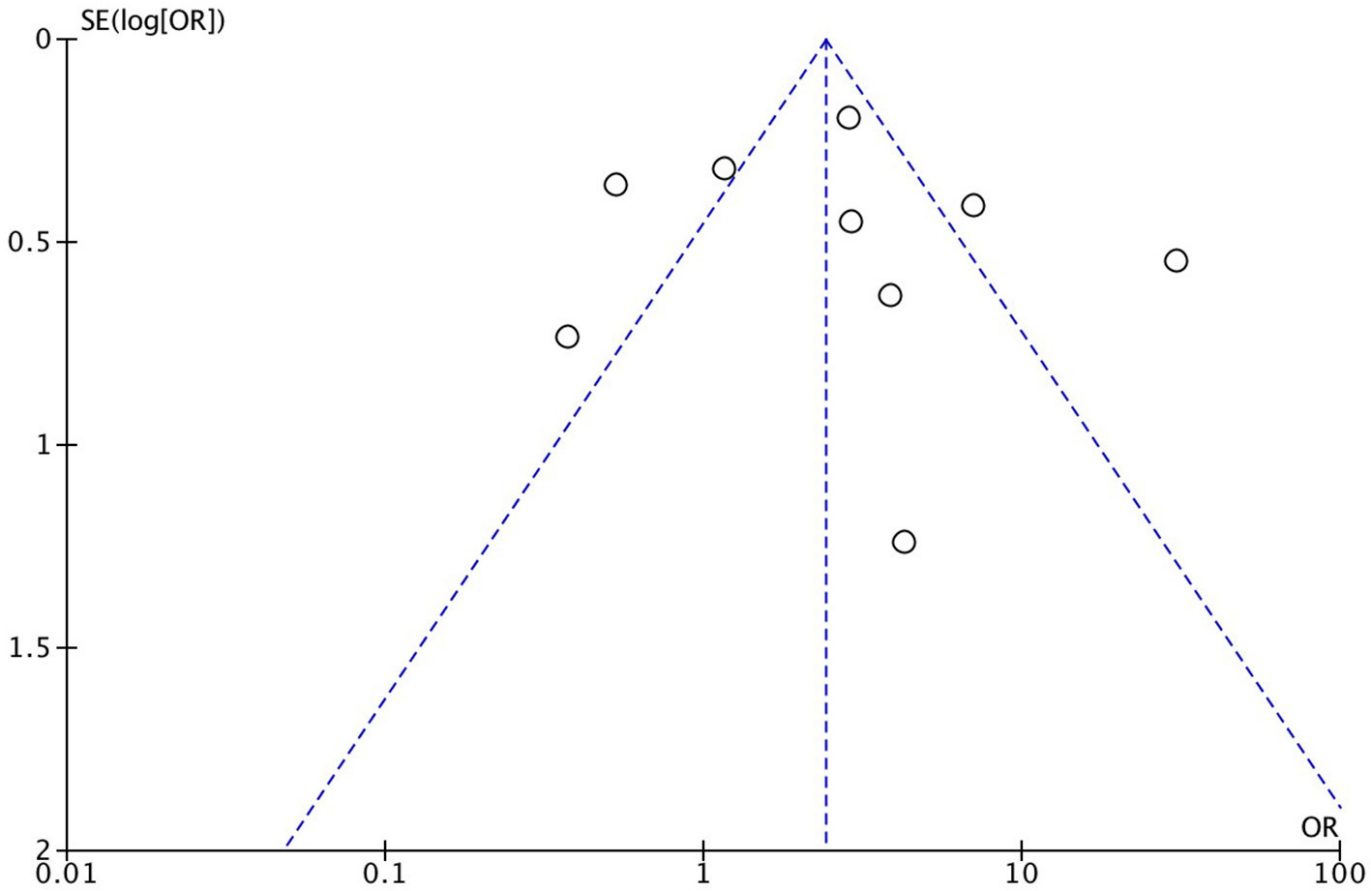
Funnel plot for total revascularization rate.

**Figure 6:**
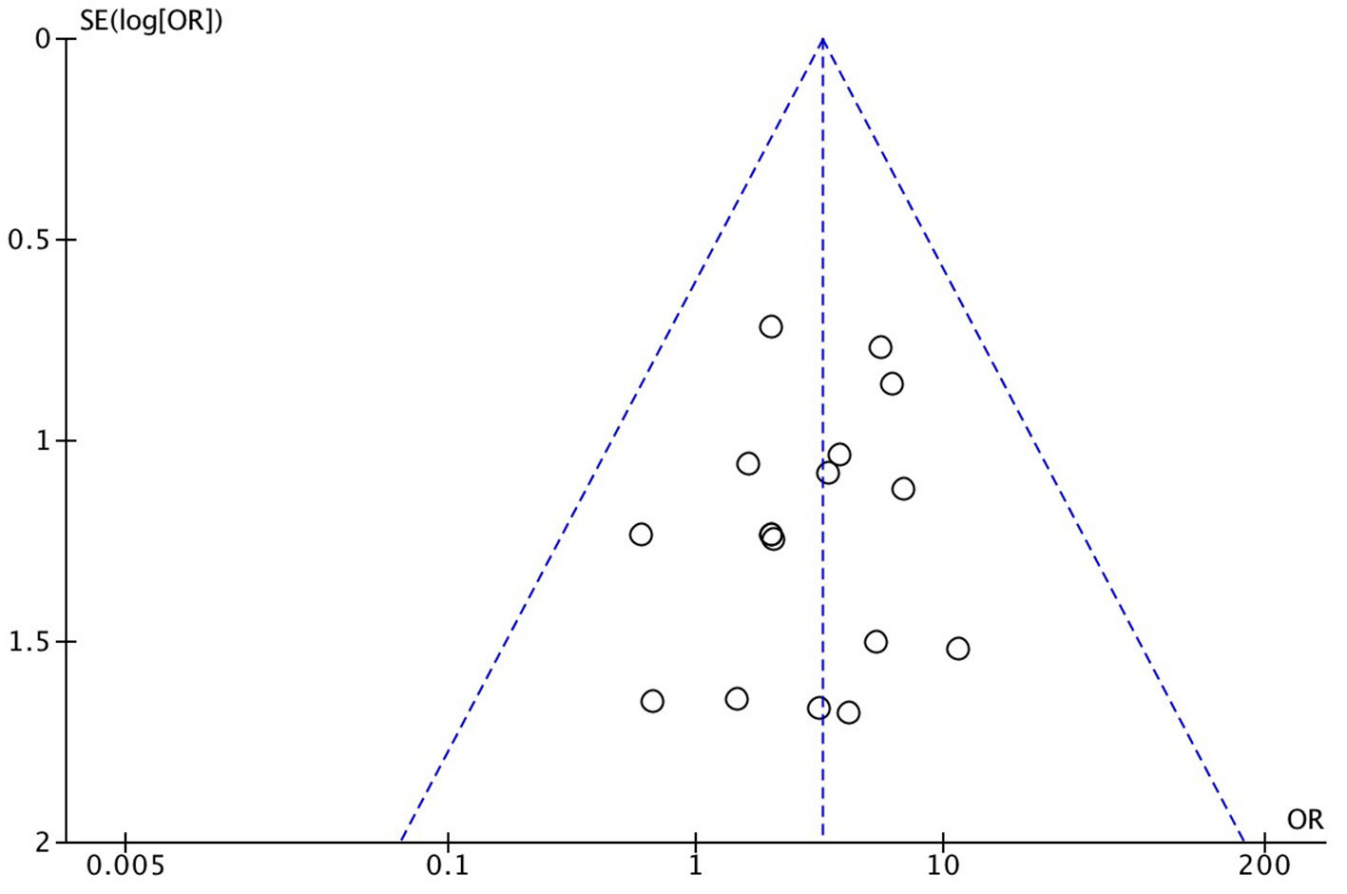
Funnel plot for perioperative neurological events.

As demonstrated in Figure 1, we identified and included 22 studies that had compared the mortality of on-pump vs. off-pump in redo CABG (8–29), and 5,197 individuals were recruited including 3,215 in on-pump group and 1,982 in off-pump group. All the studies were retrospective cohort design with medium or high quality according to Newcastle Ottawa Quality Assessment Scale (Table 1). Funnel plot was performed to assess the publication bias for every outcomes of interest (Figures 2–6) and visual inspection of the funnel plot didn't reveal significant publication bias for early mortality, mid-term mortality, total revascularization and perioperative neurological events. However, visual inspection of the funnel plot for the long-term mortality showed significant publication bias. The detailed information of the studies was listed in Table 2 and the patients demographic characteristics were shown in Table 3.

**Table 1:** Quality assessment of included studies.

**References**	**Selection**	**Comparability**	**Outcome**	**Quality scoring**
Aranda-Michel et al. (8)	4	1	3	8
Bergsland et al. (9)	4	1	2	7
Bruno et al. (10)	4	1	3	8
Czerny et al. (11)	3	1	3	7
D'Ancona et al. (12)	3	2	3	8
Dewey et al. (13)	3	1	3	7
Dohi et al. (14)	4	1	3	8
Iscan (15)	3	1	2	6
Kara et al. (16)	4	1	3	8
Mishra et al. (17)	3	2	2	7
Morris et al. (18)	3	1	3	7
Ramlawi et al. (19)	4	1	2	7
Rufa et al. (20)	4	2	2	8
Schutz et al. (21)	2	2	2	6
Shapira et al. (22)	4	1	3	8
Shin et al. (23)	3	2	3	8
Stamou et al. (24)	3	1	3	7
Teodori et al. (25)	3	1	3	7
Tugtekin et al. (26)	4	2	2	8
Usta et al. (27)	4	1	3	8
Vohra et al. (28)	4	1	3	8
Wu et al. (29)	3	1	2	6

*Quality assessment is based on the Newcastle Ottawa Quality Assessment Scale*.

**Table 2:** Studies included in the meta-analysis and quality scoring.

**References**	**Journal**	**Study design**	**Race**	**Country**	**Period**	**Sample size (redo-OPCAB/redo-CABG)**	**Quality scoring**
Aranda-Michel et al. (8)	Cardiovasc Revasc Med	Retrospective, Observational	Caucasian, Black	USA	2011–2017	350 (41/309)	8
Bergsland et al. (9)	Eur J Cardiothorac Surg	Retrospective, Observational	Caucasian	USA	1995.1–1996.12	288 (105/183)	7
Bruno et al. (10)	Eur J Cardiothorac Surg	Retrospective, Observational	Caucasian	UK	1996.5–2014.1	176 (88/88)	8
Czerny et al. (11)	Ann Thorac Surg	Retrospective, Observational	Caucasian	Austria	1995.1–2002.4	118 (44/74)	7
D'Ancona et al. (12)	Heart Surgery Forum	Retrospective, Observational	Caucasian	USA	1995.1–1999.3	581 (274/307)	8
Dewey et al. (13)	Heart Surgery Forum	Retrospective, Observational	Caucasian	USA	1998.1–2000.12	432 (153/279)	7
Dohi et al. (14)	Eur J Cardiothorac Surg	Retrospective, Observational	Asian	Japan	2008–2011	400 (200/200)	8
Iscan (15)	Cardiovasc Surg	Retrospective, Observational	Caucasian	Turkey	1978–2000	113 (32/81)	6
Kara et al. (16)	Ann Thorac Cardiovasc Surg	Retrospective, Observational	Caucasian	Turkey	1998–2010	105 (52/53)	8
Mishra et al. (17)	J Thorac Cardiovasc Surg	Retrospective, Observational	Caucasian	India	1996.1–2005.12	538 (332/206)	7
Morris et al. (18)	Innovations	Retrospective, Observational	Caucasian	USA	1997.1–2004.12	771 (132/639)	7
Ramlawi et al. (19)	Innovations	Retrospective, Observational	Caucasian	USA	2004.1–2011.7	266 (62/204)	7
Rufa et al. (20)	J Thorac Cardiovasc Surg	Retrospective, Observational	Caucasian	UK	2006.1–2015.6	216 (108/108)	8
Schutz et al. (21)	Thorac Cardiovasc Surg	Retrospective, Observational	Caucasian	Germany	—	40 (20/20)	6
Shapira et al. (22)	J Card Surg	Retrospective, Observational	Caucasian	USA	1989.7–1999.7	32 (18/14)	8
Shin et al. (23)	Korean J Thorac Cardiovasc Surg	Retrospective, Observational	Asian	Korea	1996.6–2011.10	32 (18/14)	8
Stamou et al. (24)	Ann Thorac Surg	Retrospective, Observational	Caucasian	USA	1992.4–1999.7	132 (91/41)	7
Teodori et al. (25)	J Card Surg	Retrospective, Observational	Caucasian	Italy	1994.11–1999.5	166 (54/112)	7
Tugtekin et al. (26)	Clin Res Cardiol	Retrospective, Observational	Caucasian	Germany	1998.1–2004.5	195 (35/160)	8
Usta et al. (27)	J Cardiothorac Surg	Retrospective, Observational	Caucasian	Germany	2007.1–2010.12	80 (40/40)	8
Vohra et al. (28)	Eur J Cardiothorac Surg	Retrospective, Observational	Caucasian	UK	2001.4–2006.9	86 (43/43)	8
Wu et al. (29)	Chin Med Sci J	Retrospective, Observational	Asian	China	2003.1–2013.8	80 (40/40)	6

**Table 3:** Detailed demographic characteristics of individuals in the included studies.

**References**	**Treatment**	**Age, mean ± SD or median (IQR)**	**Male (%)**	**Smoking**	**DM**	**HTN**	**Dyslipi- demia**	**Lung disease**	**Renal disease**	**PAD**	**CVA**	**Prior HF**	**EF (%)**	**EuroSCORE**	**number of grafts**	**Time to previous CABG (year)**
Aranda-Michel et al. (8)	on-pump	70.7 ± 8.8	242 (78.3%)	–	149 (48.2%)	294 (95.1%)	291 (94.2%)	79 (25.6%)	3 (1%)	96 (31.1%)	26 (8.4%)	98 (31.7%)	48.9 ± 11.8	–	–	–
	off-pump	70.7 ± 8.82	30 (73.2%)	–	27 (65.9%)	37 (90.2%)	40 (97.6%)	10 (24.4%)	1 (2.4%)	18 (43.9)	6 (14.6%)	7 (17.1%)	47.7 ± 13	–	–	–
Bergsland et al. (9)	on-pump	65.7	143 (78.1%)	28 (15.3%)	59 (32.2%)	144 (78.7%)	–	42 (23%)	2 (1%)	77 (42.1%)	18 (9.8%)	19 (10.4%)	47.4	–	–	–
	off-pump	66	78 (74.3%)	8 (7.6%)	25 (23.8%)	89 (84.3%)	–	31 (29.5%)	1 (1%)	44 (41.9%)	12 (11.4%)	15 (14.3%)	45.1	–	–	–
Bruno et al. (10)	on-pump	67.7 ± 7.5	79 (89%)	60 (71%)	23 (27%)	62 (74%)	–	8 (9%)	1 (1%)	15 (18%)	9 (11%)	–	<50% 30 (36%)	7.2 ± 2.8	2.2 ± 0.8	–
	off-pump	67.3 ± 7.7	77 (87%)	65 (78%)	19 (23%)	60 (71%)	–	10 (12%)	0 (0%)	13 (15%)	9 (11%)	–	<50% 29 (34%)	7.1 ± 2.5	2 ± 0.8	–
Czerny et al. (11)	on-pump	67.1 ± 7.7	–	–	–	–	–	–	–	–	–	–	57 ± 11	6.1 ± 2.7	–	11.1 ± 5.7
	off-pump	66.9 ± 8.9	–	–	–	–	–	–	–	–	–	–	53 ± 14	6.7 ± 2.9	–	12 ± 4.9
D'Ancona et al. (12)	on-pump	65.5 (37–85)	246 (80.1%)	–	82 (26.7%)	229 (74.6)	–	73 (23.8)	1 (0.3%)	–	32 (10.4%)	21 (6.8%)	47.8 (10–76)	–	–	–
	off-pump	66.8 (41–85)	209 (76.3%)	–	60 (21.9%)	213 (77.7)	–	80 (29.2)	6 (2.2%)	–	27 (9.9)	28 (10.2%)	47 (13–84)	–	–	–
Dewey et al. (13)	on-pump	64.4 ± 9.78	–	–	–	184 (66%)	–	–	2 (0.7%)	–	26 (9.3%)	34 (12.2%)	–	–	–	–
	off-pump	64.8 ± 10.7	–	–	–	92 (60.1%)	–	–	2 (1.3%)	–	18 (11.8%)	19 (12.4%)	–	–	–	–
Dohi et al. (14)	on-pump	68.7 ± 9.4	166 (83%)	105 (52.5%)	93 (46.5%)	160 (80%)	122 (61%)	5 (2.5%)	51 (25.5%)	37 (18.5%)	21 (10.5%)	33 (16.5%)	≤ 60% 64% <30% 8%	–	–	–
	off-pump	68.1 ± 9.3	156 (78%)	108 (54%)	94 (47%)	144 (72%)	121 (60.5%)	4 (2%)	53 (26.5%)	38 (19%)	27 (13.5%)	33 (16.5%)	≤ 60% 74% <30% 10%	–	–	–
Iscan (15)	on-pump	61.3 ± 5	67 (82.7%)	31 (40.3%)	24 (31.2%)	39 (50.7%)	35 (45.6%)	8 (10.4%)	8 (10.4%)	4 (5.2%)	–	–	–	–	–	–
	off-pump	57.4 ± 7.3	26 (81.3%)	12 (38.7%)	10 (32.3%)	14 (45.2%)	13 (42%)	4 (12.9%)	6 (19.4%)	2 (6.6%)	–	–	–	–	–	–
Kara et al. (16)	on-pump	58.11 ± 8.11	46 (86.8%)	30 (56.6%)	14 (26.4%)	47 (88.7%)	23 (43.4%)	–	–	5 (9.4%)	5 (9.4%)	–	30%−50% 18 (34%) <30% 13 (24.5%)	–	–	7.34 ± 5.54
	off-pump	59.08 ± 9.51	45 (86.5%)	35 (67.3%)	10 (19.2%)	48 (92.3%)	21 (40.4%)	–	–	10 (19.2%)	2 (3.8%)	–	30%−50% 16 (30.8%) <30% 17 (32.7%)	–	–	8.27 ± 5.27
Mishra et al. (17)	on-pump	61.2 ± 6.1	183 (88.8%)	35 (17%)	64 (31.1%)	108 (52.4%)	–	17 (8.2%)	3 (1.4%)	7 (3.4%)	5 (2.4%)	10 (4.9%)	43.1 ± 6.6	–	–	6.58 ± 1.17
	off-pump	60.4 ± 5.8	296 (89.2%)	55 (16.6%)	108 (32.5%)	159 (47.9%)	–	25 (7.5%)	5 (1.5%)	19 (5.7%)	12 (3.6%)	23 (6.9%)	42.6 ± 6.8	–	–	7.42 ± 1.42
Morris et al. (18)	on-pump	66.2 ± 9.4	545 (85.3%)	155 (24.2%)	203 (31.8%)	–	–	96 (15%)	38 (5.9%)	U/A	203 (31.8%)	111 (17.4%)	46.1 ± 12.3	–	3.7 ± 0.7	–
	off-pump	67.5 ± 10.3	104 (78.8)	64 (48.5%)	52 (39.4%)	–	–	30 (22.7%)	15 (11.4%)	U/A	52 (39.4%)	37 (28%)	45 ± 13	–	3.5 ± 0.9	–
Ramlawi et al. (19)	on-pump	67 (60–74)	–	–	73 (36%)	190 (93%)	–	–	6 (3%)	30 (14.7%)	33 (16%)	28 (14%)	55 (40–60)	–	2 (2, 3)	–
	off-pump	67 (62–76)	–	–	30 (48%)	60 (97%)	–	–	4 (6.5%)	10 (16%)	19 (31%)	8 (13%)	50 (35–60%)	–	2 (1, 2)	–
Rufa et al. (20)	on-pump	71.05 ± 5.86	90 (83%)	–	39 (36%)	–	–	7 (6.5%)	12 (11%)	9 (8.3%)	10 (9.3%)	–	30–50% 33 (30.6%)	8.8 ± 3.52	–	–
	off-pump	71.29 ± 7.39	86 (80%)	–	32 (30%)	–	–	6 (5.6%)	16 (15%)	14 (13%)	12 (11%)	–	30–50% 30 (27.8%)	9.21 ± 3.2	–	–
Schutz et al. (21)	on-pump	67.1 ± 6.6	18 (90%)	9 (45%)	5 (25%)	12 (60%)	13 (65%)	–	–	–	–	–	48.2 ± 15.3	–	–	–
	off-pump	63.2 ± 9.3	15 (75%)	6 (30%)	2 (10%)	14 (70%)	11 (55%)	–	–	–	–	–	52.8 ± 13.9	–	–	–
Shapira et al. (22)	on-pump	67 ± 9	12 (85.7%)	4 (29%)	7 (50%)	11 (79%)	–	–	–	5 (36%)	5 (36%)	–	43 ± 13	–	–	–
	off-pump	65 ± 8	14 (77.8%)	7 (39%)	7 (39%)	17 (94%)	–	–	–	6 (33%)	6 (33%)	–	46 ± 15	–	–	–
Shin et al. (23)	on-pump	64.3 ± 8.1	9 (64.2%)	5 (35.7%)	4 (28.6%)	12 (85.7%)	4 (28.6%)	–	1 (7.1%)	0	1 (7.1%)	–	EF <35% 3 (21.4%)	8.5 ± 2.4	–	–
	off-pump	65.5 ± 7.2	12 (66.7%)	5 (27.8%)	8 (44.4)	10 (55.6%)	5 (27.8%)	–	1 (5.6%)	4 (24.3%)	3 (16.7%)	–	EF <35% 2 (11.1%)	7.4 ± 2.0	–	–
Stamou et al. (24)	on-pump	65 ± 9	25 (61%)	–	–	–	–	–	–	–	–	–	EF <35% 18 (44%)	–	–	–
	off-pump	65 ± 10	66 (72%)	–	–	–	–	–	–	–	–	–	EF <35% 39 (43%)	–	–	–
Teodori et al. (25)	on-pump	62.7 ± 8.6	100 (89.3%)	–	–	–	–	–	–	18 (16.1%)	10 (8.9%)	1 (0.9%)	–	–	–	9.38 ± 5
	off-pump	64.7 ± 8.5	48 (88.9%)	–	–	–	–	–	–	7 (12.9%)	1 (1.8%)	2 (3.7%)	–	–	–	10 ± 4.7
Tugtekin et al. (26)	on-pump	66 ± 8.1	132 (82.5%)	–	59 (36.8%)	–	–	10 (6.2%)	–	26 (16.3%)	–	–	55 ± 16.2%	–	–	7.99 ± 4.9
	off-pump	66.9 ± 7.9	28 (53.8%)	–	12 (34.3%)	–	–	2 (5.7%)	–	7 (20%)	–	–	52 ± 14.4%	–	–	7.93 ± 5.0
Usta et al. (27)	on-pump	71 ± 9	36 (90%)	61 ± 5%	16 (40%)	38 (95%)	–	–	–	–	–	–	–	–	–	11.58 ± 5.3
	off-pump	72 ± 10	32 (80%)	65 ± 48%	17 (43%)	40 (100%)	–	–	–	–	–	–	–	–	–	11.67 ± 5.7
Vohra et al. (28)	on-pump	64.7 ± 7.7	38 (88.3%)	U/A	14 (32.5%)	34 (79%)	36 (83.7%)	5 (11.6%)	1 (2.3%)	4 (9.3%)	–	–	EF <30% 14 (32.5%)	5 ± 3.4	–	–
	off-pump	65.7 ± 6.9	41 (95.3%)	–	10 (23.2%)	27 (62.8%)	39 (90.7%)	4 (9.3%)	1 (2.3%)	4 (9.3%)	–	–	EF <30% 19 (44.1%)	5 ± 4.7	–	–
Wu et al. (29)	on-pump	60.4 ± 8.79	32 (80%)	15 (37.5%)	21 (52.5%)	23 (57.5%)	28 (70%)	2 (5%)	1 (2.5%)	8 (20%)	2 (5%)	–	49 ± 26	–	–	–
	off-pump	62 ± 7.38	29 (72.5%)	20 (50%)	18 (45%)	19 (47.5%)	31 (77.5%)	10 (25%)	9 (22.5%)	11 (27.5%)	1 (2.5%)	–	43 ± 10	–	–	–

*DM, diabetes mellitus; HTN, hypertension; PAD, peripheral artery disease; CVD, cerebral vascular disease; HF, heart failure; EF, ejection fraction*.

**Figure 7:**
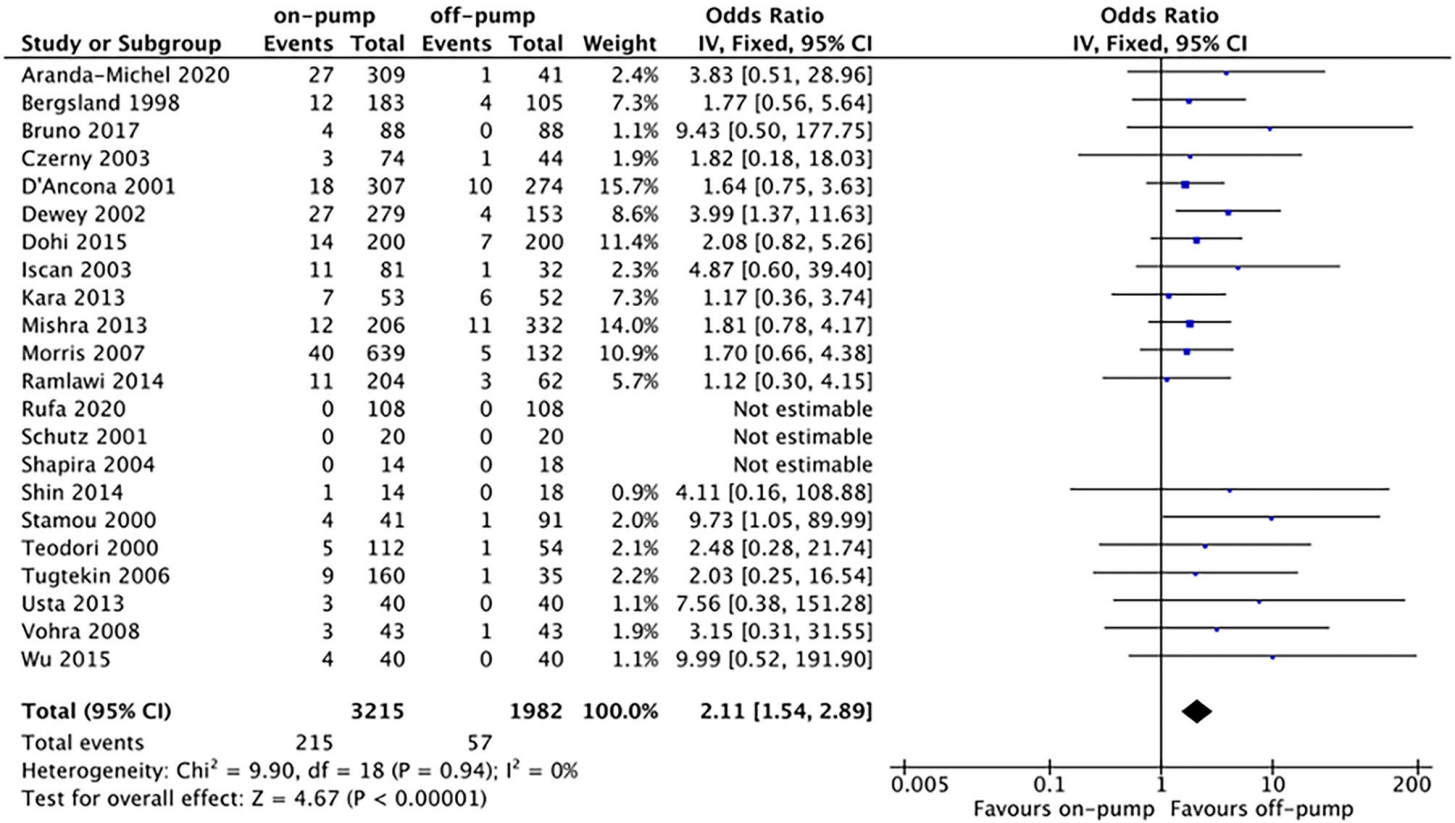
Forest plot for early mortality.

**Figure 8:**
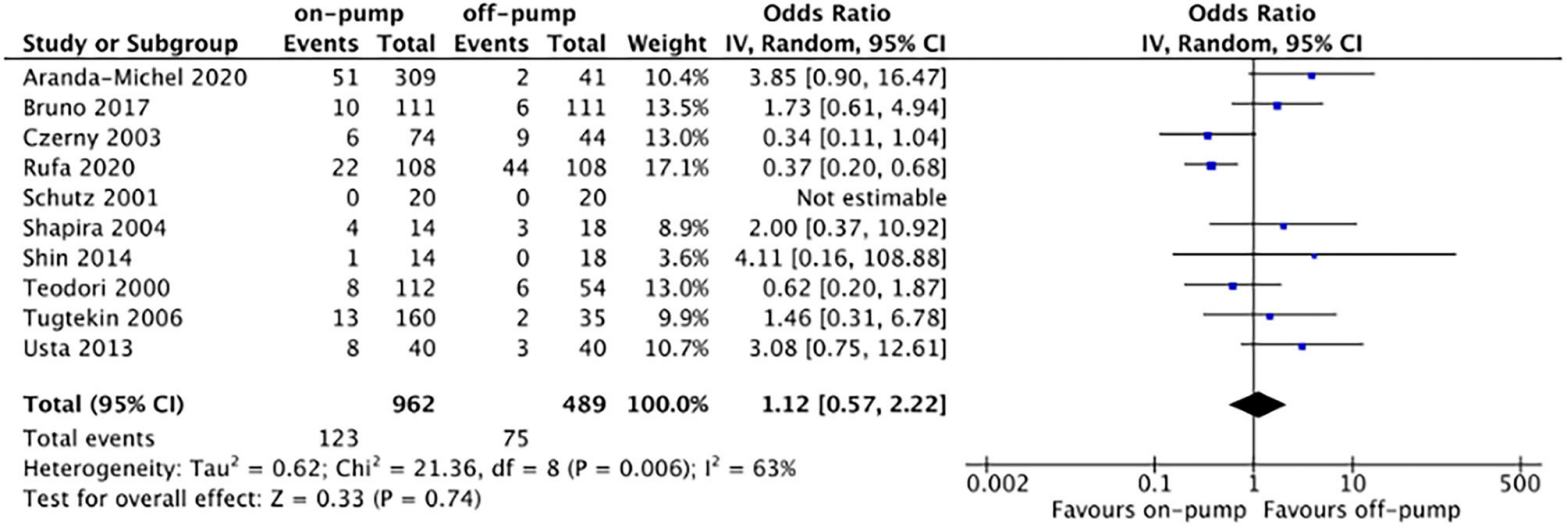
Forest plot for mid-term mortality.

**Figure 9:**
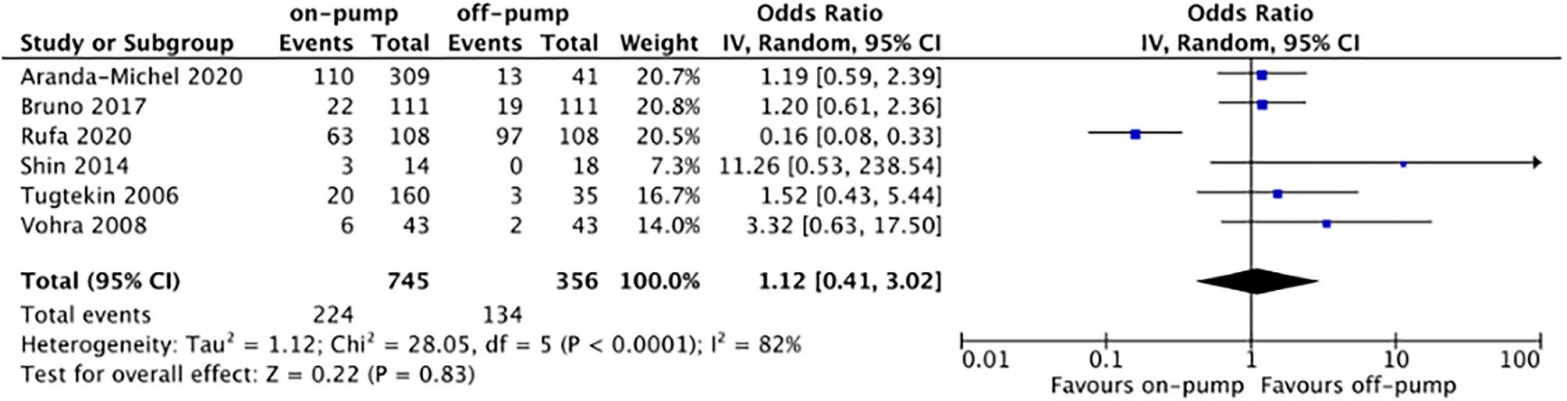
Forest plot for long-term mortality.

**Figure 10:**
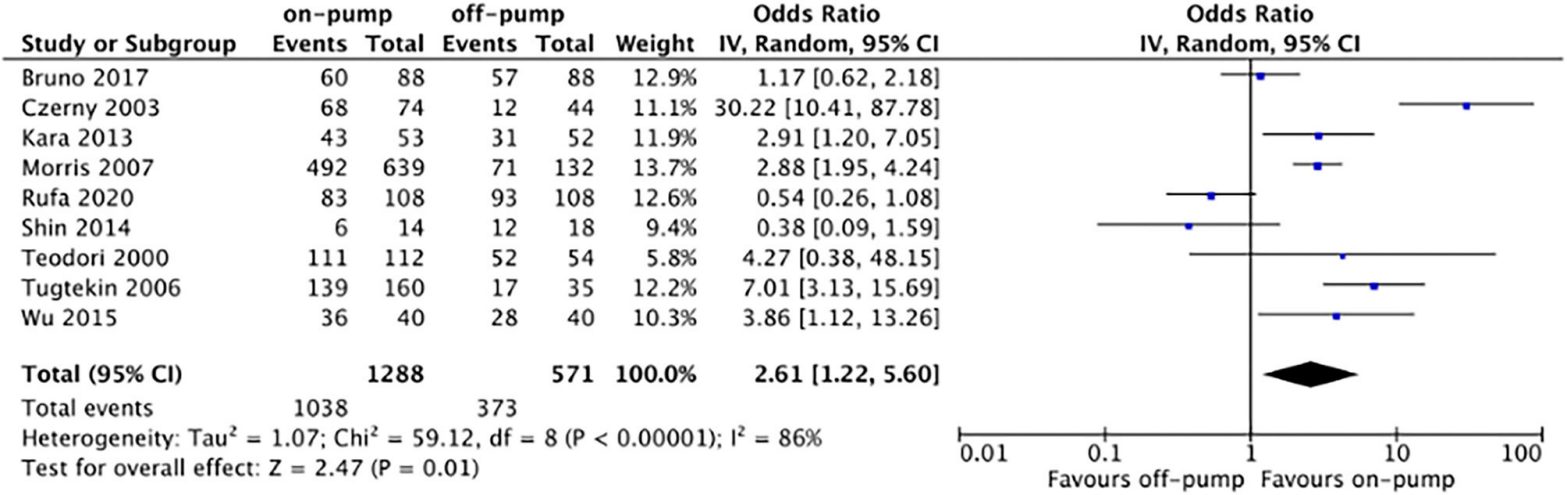
Forest plot for total revascularization rate.

**Figure 11:**
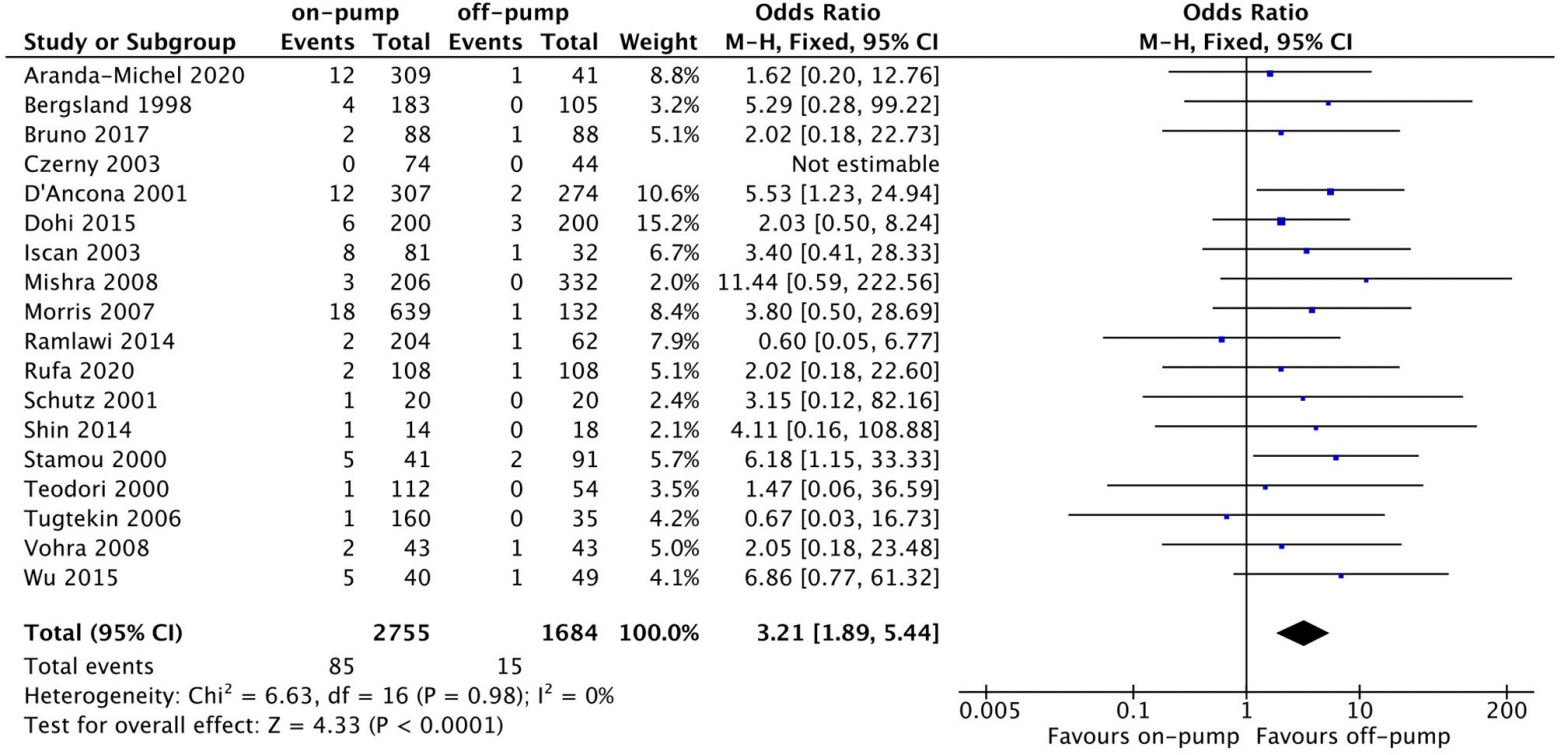
Forest plot for perioperative neurological events.

Compared with off-pump redo CABG, on-pump technique was associated with significantly higher early mortality rate (OR 2.11, 95%CI: 1.54–2.89, Z = 4.67, *P* < 0.00001, Figure 7) with low heterogeneity (*I*^2^ = 0%, *P* = 0.94). There was no difference between on-pump redo CABG group and off-pump redo CABG group in term of mid-term (OR 1.12, 95%CI: 0.57–2.22, Z = 0.33, *P* = 0.74, Figure 8) with moderate heterogeneity (*I*^2^ = 63%, *P* = 0.006). Similarly, no significant difference was noted between on-pump redo CABG group and off-pump redo CABG group in term of long-term mortality (OR 1.12, 95%CI: 0.41–3.02, Z = 0.22, *P* = 0.83, Figure 9) with high heterogeneity (*I*^2^ = 82%, *P* < 0.0001). The rate of total revascularization was higher in the on-pump redo CABG group than off-pump redo CABG group (OR 2.61, 95%CI: 1.22–5.60, Z = 2.47, *P* = 0.01, Figure 10) with high heterogeneity (*I*^2^ = 86%, *P* < 0.00001). The rate of perioperative neurological events was higher in the on-pump redo CABG group than off-pump redo CABG group (OR 3.21, 95%CI: 1.89–5.44, Z = 4.33, *P* < 0.0001, Figure 11) with low heterogeneity (*I*^2^ = 0%, *P* = 0.98).

To assess the impact of individual study on the pooled result, we performed a sensitivity analysis by excluding one study from the analysis (leave-one-out meta-analysis) and found that exclusion of any single study didn't substantially alter the pooled results of early, mid-term and long-term mortality between the two groups.

## Discussion

Redo CABG is a therapy for those who underwent CABG and suffered from recurred pectoris angina afterwards. However, the proportion of repeated CABG increasingly declined over time in the surgical coronary revascularization due to multiple factors, such as improved medical management, expanded application of PCI for patients after CABG, more arterial conduits in the primary CABG and so on (30). Despite improvement in the pre-, intra- and post-operative management in the redo CABG, mortality remains much higher than that in primary CABG. According to the STS risk model, mortality rate in redo CABG is about 3 times that of primary CABG (31). Thus, it is of great significance to improve clinical practice to lower the mortality of redo CABG. Whether utilization of cardiopulmonary machine or not is definitely an issue to be considered in the surgical revascularization of these patients. 2018 ESC/EACTS guidelines on myocardial revascularization suggest that redo CABG is preferred for patients with extensively diseased or occluded grafts, especially in the absence of patent arterial grafts (32). However, no recommendation was given on whether on-pump or off-pump is more suitable in this clinically special scenario. Considering the vacuum of recommendation in the guidelines, our study may give some implication in this aspect.

The results of the present meta-analysis demonstrate that on-pump redo CABG is associated with higher early mortality but no significant difference was noticed in mid-term or long-term mortality between on-pump redo CABG and off-pump redo CABG despite higher rate of total revascularization in on-pump group. Echoing results were reported in previous meta-analysis by Zhang et al. (33) that the off- pump technique was associated with significantly reduced 30-day mortality (OR=0.43, 95% CI 0.26–0.72, *p* = 0.001). But it didn't analyze mid-term or long-term mortality. Sepehripour et al. reported similar mid-term mortality of on-pump redo CABG vs. off-pump redo CABG (OR=1.07, 95% CI 0.58–1.96, *p* = 0.73) in their meta-analysis in 2013 but lacked long-term mortality analysis (34). In addition, apart from the studies published before 2010 and included in the meta-analysis by Sepehripour, we added another 9 studies (8, 10, 14, 16, 19, 20, 23, 27, 29) including 1705 individuals published after 2010 to our meta-analysis. In comparison with two early meta-analysis (33, 34) comparing mortality between on-pump and off-pump redo CABG, we included much more recent studies and shed light on long-term mortality (≥5 years) as well as early (perioperative period, in hospital or 30-day) and mid-term mortality (≥1 year and <5 year). Notably, we recognized higher rate of perioperative neurological events in the on-pump group which was similar to the previous findings in the first CABG (35). Those who are older (36) and with cerebrovascular disease (37) may benefit much more from off-pump technique. Considering redo CABG patients are generally older and have higher prevalence of cerebrovascular disease than primary CABG patients, off-pump technique may be more appropriate in term of reducing perioperative neurological risk.

Several factors may explain the difference in early mortality between the two groups. First, all the included studies were retrospective cohort study which led to the mismatch of baseline characteristics between the two groups. The severity of patient medical condition in on-pump group is more serious than that in the off-pump group. Aranda-Michel et al. tended to refer patients to the on-pump redo CABG if the patients had heart failure within 2 weeks or had cardiogenic shock (8). Usta et al. noticed higher number of distal anastomosis in the on-pump group which meant more coronary or graft lesions (27). Mishra listed the factors encouraging surgeons to select on-pump technique as worse hemodynamic status, urgency of the operation and worse quality of distal coronary target vessels (17). Second, the inadequate myocardial protection may increase the risk of perioperative myocardial infarction. The extensive coronary and graft stenosis or occlusion could lead to the uneven distribution of cardioplegic solution and subsequently incur the myocardial injury in the redo coronary surgery with cardiopulmonary bypass. Tugtekin et al. established perioperative myocardial infarction as an independent predictor for hospital mortality (26). Third, avoidance of aorta manipulation in off-pump redo CABG might reduce the risk of stroke due to cerebral embolism. Our study also showed that the perioperative neurological risk in on-pump group was about 3 times that in the off-pump group. Several studies utilized anastomosis assist device to perform anastomosis so as to completely avoid “side-biting” clamps in redo off-pump CABG and reduce the incidence of stroke (19, 29). Fourth, several minimally invasive thoracotomy applied during off-pump redo CABG could alleviate the surgical trauma, minimize dissection of the heart as well as previous graft, reduce bleeding and avoid re-sternotomy which potentially injured the previous grafts beneath the sternum (22, 24).

In our meta-analysis, despite survival advantage of off-pump technique in the early period after surgery, no significant difference was detected in the mid- and long-term mortality between the two groups. In other words, the early survival advantage of off-pump redo CABG didn't persist into mid- and long-term period. It is generally acknowledged that completeness of revascularization is the key parameter associated with long-term outcomes. The results of our meta-analysis demonstrated the proportion of complete revascularization in off-pump group was significantly lower than that in on-pump group. However, the lower complete revascularization rate in the redo CABG may not significantly influence the long-term mortality. As Kara et al. postulated, the revascularization of the left anterior descending artery is the primary factor in the long-term survival (16). Following the CABG, total revascularization could be achieved with PCI, which is known as hybrid revascularization (38). Bilal et al. matched the two groups according to the baseline characteristics including extent of coronary disease using propensity score matching but noticed lower number of grafts in the off-pump group without different reintervention rate between the two groups (39). Thus, they contended that it was the matter of a tendency to overgraft in the on-pump CABG rather than incomplete revascularization in the off-pump CABG. In addition to complete revascularization, the graft patency plays a pivotal role in the long-term outcomes as well. Compared with on-pump redo CABG, more arterial grafts, which presented higher patency than vein grafts, were used in off-pump redo CABG (10, 20, 23, 27). It was shown by Schuts that in the mean follow up period of 22 months, the postoperative graft patency rate was 95% as confirmed by angiography in the off-pump redo CABG group (21). Usta applied SF-36 questionnaire consisting of 36 questions in eight areas to assess patients' quality of life after surgery and found that there was no significant difference in quality of life between the two groups (27).

To date, this is the first meta-analysis comparing long-term mortality between on-pump redo CABG and off-pump redo CABG. It provides a quantitative summary of the available evidence surrounding the use of off-pump technique in redo CABG and may provide some clinical implication for clinical practitioners.

With the advance of cardiac surgery, surgeons are persistently pursuiting less invasive surgical techniques, among which off-pump technique plays an important part as well as other minimally invasive techniques. The technique of off-pump yields superior early survival, lower neurological risk and comparable mid-term and long-term survival compared to on-pump technique. This indicates that off-pump technique may serve as a viable option for patients requiring redo CABG, especially those high-risk patients.

Several limitations shouldn't be neglected about this meta-analysis. Firstly, all the studies included in the meta-analysis were retrospective cohort study and our findings may be biased by the retrospective, non-randomized nature of the studies. However, due to the small volume of redo CABG patients and those complex clinical conditions, these individuals may not be subject to randomized controlled trials. Secondly, the baseline risk profile differed between on-pump and off-pump groups. Patients who had more coronary or graft vessels lesions and needed more grafts tended to be operated with on-pump techniques, which revealed selection bias. Finally, most of the included studies were from institutions with a wealth of experience on off-pump technique, and this might limit the applicability of the findings to institutions with less proficiency in off-pump CABG.

In conclusion, despite lower rate of complete revascularization, off-pump redo CABG was associated with superior early survival to on-pump redo CABG and lower perioperative neurological risk, while no significant difference was noticed in the mid-term and long-term mortality between the two groups. So off-pump technique is a safe and effective alternative to on-pump technique in redo CABG, especially in those with high-risk profile. However, high risk of selection bias should be noted and this may influence the results. Further high-quality trials are warranted to decide whether off-pump technique could confer better results in redo CABG patients. For the sake of least invasiveness and best outcomes, surgeons are supposed to weigh the pros and cons of cardiopulmonary bypass in redo CABG and make the individualized decision for patients.

## Data Availability Statement

The original contributions presented in the study are included in the article/supplementary material, further inquiries can be directed to the corresponding author.

## Author Contributions

WF contributed to the conception of the research idea, supervision of the study, and validation of the results. SZ collected relevant data, performed analysis, and wrote the original draft. SH collected relevant data and provided assistance in manuscript revision. YS collected relevant data. XT reviewed and edited the manuscript. All authors contributed to the article and approved the submitted version.

## Funding

This work was supported by the National Key Research and Development Program [2018YFC1311201] from the Ministry of Science and Technology of People's Republic of China.

## Conflict of Interest

The authors declare that the research was conducted in the absence of any commercial or financial relationships that could be construed as a potential conflict of interest.

## Publisher's Note

All claims expressed in this article are solely those of the authors and do not necessarily represent those of their affiliated organizations, or those of the publisher, the editors and the reviewers. Any product that may be evaluated in this article, or claim that may be made by its manufacturer, is not guaranteed or endorsed by the publisher.
